# Social network correlates of IPV acceptance in rural Honduras and rural Uganda^☆^

**DOI:** 10.1016/j.ssmph.2018.02.001

**Published:** 2018-02-06

**Authors:** Holly B. Shakya, Jessica M. Perkins, Margaret Traeger, Alexander C. Tsai, David R. Bangsberg, Bernard Kakuhikire, Nicholas A. Christakis

**Affiliations:** a Center on Gender Equity and Health, School of Medicine, University of California San Diego, San Diego, CA, United States; b Department of Human and Organizational Development Peabody College, Vanderbilt University, PMB 90, 230 Appleton Place, Nashville, TN 37203, United States; c Department of Sociology, Yale Institute for Network Science, P.O. Box 208263, New Haven, CT 06520-8263, United States; d Harvard Center for Population and Development Studies, Harvard University, 9 Bow St, Cambridge, MA 02138, United States; e School of Public Health, Oregon Health and Science University and Portland State University, MC: GH230 3181 SW Sam Jackson Park Road, Portland, OR 97239, United States; f Mbarara University of Science & Technology, Mbarara, Uganda

**Keywords:** Honduras, Uganda, Social networks, Social norms, Intimate partner violence, Social cohesion

## Abstract

We investigated the household-level social network correlates of acceptance of intimate partner violence (IPV) in rural, agrarian settings of Honduras and Uganda, two low-income countries with unequal access to resources based upon gender. We collected complete social network data in each location (Honduras in 2014 and Uganda in 2012), across a diverse range of relationships, and then created a measure of household cohesion by calculating the degree to which members of a household nominated each other as important social connections. Our measure of IPV acceptance was based on 4 questions from the Demographic Health Survey to assess the conditions under which a person believes that it is acceptable for a man to perpetrate physical violence against his wife or partner and we coded a person as positive on IPV acceptance if they answered positively to any of the four questions. We used logistic regression to calculate the odds that an individual accepted IPV given (1) household level cohesion and (2) the proportion of the household that accepts IPV. We found individuals from more cohesive households were less likely to accept IPV controlling for the overall level of IPV acceptance in the household. Nevertheless, those in households more accepting of IPV were more likely to personally accept IPV. In stratified analyses, when household IPV acceptance was especially high, the benefit of household cohesion with respect to IPV was attenuated. The design and implementation of interventions to prevent IPV should consider household structure and norms rather than focusing only on individuals or couples.

## Highlights

• Attitudes towards intimate partner violence are associated with its perpetration.
• Social cohesion has been shown to be protective against violence.
• We look at household cohesion and violence acceptance in Honduras and Uganda.
• Those within socially cohesive household are less likely to accept partner violence.
• This finding depends on the level of violence acceptance within the household.

## Introduction

Globally, approximately 30% of women who have ever been in an intimate relationship have reported physical or sexual violence by an intimate partner (WHO, 2013). There is a growing body of evidence that intimate partner violence (IPV), and attitudes accepting of IPV, are socially clustered, supported by community and family social practices, and transmitted through families (Shakya et al., 2016, Shakya et al., 2017). For example, people who have witnessed IPV as children are more likely to experience or perpetrate it as adults (Sambisa, Angeles, Lance, Naved, & Thornton, 2011), and female victims of IPV are more likely to report attitudes accepting of IPV (Hindin, Kishor, & Ansara, 2008b). While previous research has inferred social clustering of IPV acceptance through individual-level questions or aggregated measures at the level of states or other area units, few studies have used social network data to investigate these behavioral and attitudinal clusters.

### Conceptual model

The pattern of social ties in which a person is embedded, and the normative beliefs and practices of those to whom s/he is connected, may clearly affect an individual’s beliefs and practices. In social norms theory, *reference groups* are those to whom an individual turns for information on the expected ways of behaving within group-specific contexts (Ajzen and Fishbein, 1973, Shakya et al., 2014). *Descriptive norms* are the behaviors commonly practiced in a group, and are supported through observation of what the majority of others are doing (or not doing) (Cialdini, Reno, & Kallgren, 1990). *Injunctive norms,* on the other hand, reflect the expectations of the community, and are often reinforced through perceived social or individual consequences in the form of sanctions. Because of the threat of sanctions, the proscribed behavior may rarely be observed, making it difficult to ascertain whether the behavior is simply uncommon or is actually against an underlying social norm (Borsari & Carey, 2003). Sanctions can be positive for compliance, and can include social rewards such as approval or inclusion in social groups. They can also be negative for non-compliance, and may be as overt as stoning or as subtle as quiet disapproval, or may simply consist of the withholding of social rewards (Bell & Cox, 2015).

Social network analysis is a powerful tool for investigating social norms among specific groups because it can identify the people to whom individuals are most closely connected and these people’s salient characteristics. For example, previous work on latrine adoption in India demonstrated the social clustering of behaviors through social network analysis as well as the positive relationship between injunctive norms and the level of connection within a community (Shakya et al., 2014). There may be multiple reference groups for any given behavior, and social network analysis can be used to at least partially identify those groups and the levels and directions of their influence. The ability of social network analysis to identify these groups, however, will depend upon the questions used to elicit the social networks, the utility of those questions in capturing the relevant relationships, and the scale of the network study (Shakya et al., 2016, Shakya et al., 2017).

While a community may be opposed to IPV, families within that community may accept it (Shakya et al., 2016). If there are injunctive norms at the community level against perpetrating IPV, but IPV is occurring within families, then family-level norms may contribute to its continuation (Shakya et al., 2016). The family may be at least one of the reference groups to which individuals (subconsciously or consciously) turn for information on behavioral expectations regarding IPV. IPV is as an example of a practice that, because it often takes place in the privacy of the home, is generally less detectable than practices such as child marriage. With such practices, the varying influences of different reference groups is particularly important. Previous research has in fact pointed towards the possibility of an “inner norm” within the family that is supportive of IPV, versus an “outer norm” within the community that opposes it (Shakya et al., 2016). Thus, family-level characteristics, as opposed to those within the greater community, can offer important insights about factors that contribute to IPV in different contexts. However, families cannot easily change their views or practices if community norms are against it, as in the case of female genital cutting (Vogt, Mohmmed Zaid, El Fadil Ahmed, Fehr, & Efferson, 2016).

Research on violence has shown that family cohesion, defined as emotional support and positive communication, can be protective against violence among youth (Gorman-Smith, Henry, & Tolan, 2004), possibly by providing protection against social stress, an environment of security, strong parental monitoring, and positive family communication (Kliewer et al., 2004). Consistent with the findings on family cohesion, research on community-level violence has also identified community social cohesion as an important factor in violence prevention (Kennedy, Kawachi, Prothrow-Stith, Lochner, & Gupta, 1998). While cohesive communities may protect against violence by providing a warm and nurturing environment, it is also hypothesized that more socially cohesive communities are able to maintain social control through the creation and maintenance of injunctive norms which can be used effectively to discourage violence (Kennedy et al., 1998). This sort of control, however, can also be effective at encouraging violence in contexts in which violence is acceptable and normative. Literature on social networks has demonstrated that cohesion can reinforce norms, whether positive or negative (Centola and Macy, 2007, Latkin et al., 2003). Thus, understanding the association between cohesion and acceptance of violence, and the possible mechanisms by which this association occurs, is an important question in research on family-level violence prevention.

Despite the evidence on the relationship between violence and social cohesion, few studies have considered family cohesion in the context of IPV (Olsen & Lovett, 2016). Furthermore, the majority of cohesion research has operationalized cohesion using survey questions that ask respondents to report on the quality of their interactions within their families or communities, which can be subject to response bias depending upon who within the community or the family is being asked the questions. For instance parents are more likely to report positive parent-child interactions than are children (Steinberg, Lamborn, Darling, Mounts, & Dornbusch, 1994).

Full social network data in developing countries is rare (Perkins, Subramanian, & Christakis, 2015). Here, we combine two very uncommon social network datasets, one from rural Honduras and one from rural Uganda, to investigate the household-level social network correlates of IPV acceptance. Although Honduras and Uganda represent distinct geographic settings, both countries represent low-income, low-resource societies with strong patriarchal cultural traditions (Hernandez, 2002, Kafumbe, 2010). In addition, both countries have a history of societal violence (Kasozi, 1994, Briceño-León et al., 2008) and exhibit strongly unequal gender norms (Mirembe and Davies, 2001, Godfrey, 2010, Lomot, 2013). Past studies have shown some of these factors and others (alcohol consumption, limited social support, gender inequality, witnessing violence as a child) to be associated with IPV (Garcia-Moreno, 2005, Kwagala et al., 2013).

We investigate the extent to which social network factors at the individual and household level are associated with individual attitudes accepting of IPV. We hypothesize that individuals from more cohesive households will be less likely to accept IPV; distinctly, we hypothesize that individuals in households in which a greater proportion of household members accept IPV will be more likely to accept IPV. We will also consider the interaction between these two: is household cohesion more strongly associated with IPV acceptance in households in which IPV acceptance is higher overall? Finally, given that IPV acceptance can differ according to education, gender, and marital status (Shakya et al., 2016), we consider the proportion of the household that is male, the proportion of the household that is married, and the mean level of household education separately, as possible factors associated with individual IPV attitudes accepting of IPV.

## Methods

### Data collection

In 2014, we collected full sociocentric network data from individuals aged 13+ in two villages in *La Unión, Lempira*, Honduras, as part of a larger ongoing study (Shakya, Stafford et al., 2017). Sociocentric studies attempt to ascertain all of the social relationships within a defined population (Marin & Wellman, 2011). Although adolescents 13–17 years of age were surveyed, we eliminated their observations to maintain consistency with the Uganda sample. In each Honduran village, we took a complete census of all households, which included mapping each household in the village and enumerating all of the residents within them. We later returned to each household to gather data about individual health indicators, attitudes and beliefs, demographics, and social network connections. In total, our Honduras household census revealed a population of 1307 eligible individuals, and we were able to collect survey and network data on 837 (64%) individuals (691 adults after excluding adolescents).

In each of eight villages within one parish in rural southwest Uganda, a data collection procedure similar to that used in Honduras was implemented based on a complete census of all adults aged 18 years and above. A total of 1669 people (out of 1747 residents or 96%) were interviewed in 2011 and 2012 across 716 households. In both Uganda and Honduras, households in which only one individual was surveyed were excluded from the analyses (Honduras N = 43, Uganda N = 172). Thus, the total sample size from Honduras was 691 and the total sample size from Uganda was 1392.

### Social ties

A “name generator” is a question asked of a respondent to elicit important social connections (Shakya, Christakis et al., 2017). In both Uganda and Honduras, participants were asked a series of questions to elicit the names of important social connections along a variety of domains. For this analysis, we were interested in ascertaining the networks that most effectively captured affective support. The challenge was that each dataset included different questions, specific to the population under consideration. Our previous research has suggested that the use of one name generator can bias the network, while too many can generate networks irrelevant to the question at hand (Shakya, Christakis et al., 2017). To determine the name generators most appropriate for our analyses, we ran factor analyses on each dataset, looking for groupings of name generators that would form one coherent cluster of questions. Factor analyses identified coherent factors for name generators in both Honduras and Uganda.

Name generators in Honduras measured emotional support by asking participants to whom they go to discuss important matters, and who they trust to discuss something personal and private: 1. What is the name of a person with whom you discuss important matters? 2. What is the name of a person that you could trust to talk about something personal or private? Respondents were told that answer choices could include friends, family, people you work with, people who work for you, neighbors, etc. For each name generator, respondents were asked to nominate up to five individuals. In Uganda, respondents were asked separately: With whom the respondent discusses financial matters, discusses health issues, and goes to for emotional support. Respondents were told to name adults who lived within the parish (in any of the eight villages) and that nominations could be repeated across questions. 1. Over the last 12 months, with whom in this parish have you usually talked about any kind of financial issues? This may include conversations about school fees, employment, giving, receiving, or paying loans, starting businesses, financing for big events, or other issues. 2. Over the last 12 months, with whom in this parish have you usually talked about any kind of health issues? This may include topics like your child’s health, family planning, nutrition, HIV, mental health, immunizations, sanitation methods, alcohol abuse or other issues. 3. Over the last 12 months, whom in this parish have you gone to for emotional support? This may include talking about both positive and negative topics such as deaths, marriages, births, loss of job, or other topics of emotional importance for you.

Questions that were not used for the analysis in Honduras included questions about who the person seeks help with a medical emergency, who they borrow and lend money to, and who they sit with at church. The three questions that were not used in Uganda were to whom they would give a honey stick, with whom they exchange food, and with whom they socialize.

### Measures

We used four questions from the DHS to assess the conditions under which a person believes that it is acceptable for a man to perpetrate physical violence against his wife or partner (Statcompiler). The questions asked: “*In your opinion, is a husband/companion justified in hitting or beating his wife/companion in the following situations:* (a) *If she leaves the house without telling him?* (b) *Neglects the children?* (c) *Argues with him?* (d) *Burns the food?* Answer choices were either yes or no. We coded a person as positive on IPV acceptance if they answered positively to any of the four questions. Cronbach’s alpha on the full scale was 0.82 for the Honduras sample and 0.71 for the Uganda sample (Shakya et al., 2016).

In this study, we calculated a measure of density for each household. Density is a measure that captures the cohesiveness of a network, and is calculated by dividing the total number of observed ties by the total number of possible ties. To do so, we identified all of the respondents within each household included in the study, and the nominated social ties amongst those individuals across all name generators. Higher density households had many individuals within the household who nominated other individuals within the household as social contacts; lower density households had few household members nominate other individuals within the household as social contacts.

Household-level measures also included the proportion of people in the individual’s household who accepted IPV, the proportion of people in the individual’s household that were male, mean household education, the proportion of the people in the individual’s household that were married or in a civil union, and the number of individuals living within each household. Household-level proportions were calculated to exclude the respondents’ own values on each measure.

Finally, we measured several individual-level factors including age, gender, education, income, religion, marital status, and, in Uganda, ethnic group. Our measure of respondents’ education was a continuous measure based on 9 categories of schooling, including early primary, primary, 4 levels of secondary, tertiary, university and postgraduate. We measured respondents’ income by asking “How much income did you personally earn from all economic activities in the past month (include farm, wage, and business work)?” Both income and education were included in the models as continuous variables.

To maintain consistency across datasets, we created standardized mean-centered measures for proportion of household that reports accepting IPV, household density, individual’s proportion of network ties that are same household, proportion of household that is male, proportion of household that is married, and household mean level of education, plus individual education and income. Mean centering was done at the level of the country dataset. As ethnic group was a relevant covariate for the Uganda dataset but was not measurable in Honduras, we included a “Honduras” ethnic group for all Honduras observations. All other measures were comparable across datasets and so left as they were. Finally, we combined the two datasets, adding in a separate measure for country. After removing observations with missing data, we had 829 respondents in Honduras and 1395 respondents in Uganda.

### Statistical analyses

We used logistic regression to estimate the relationship between individual and household characteristics and the probability of expressing acceptance of IPV, including village level fixed effects to account for village level clustering.

## Results

### Summary statistics

Table 1 shows the summary statistics for both study populations. Average age was 34 years in Honduras and 38 years in Uganda. Slightly less than half of each population was male. The average number of people interviewed per household was 3.9 in Honduras and 3.6 in Uganda. In Uganda, 30% of respondents accepted IPV; in Honduras, 24% of respondents accepted IPV. Honduran households had a lower density (0.22 (SD 0.18)) than those in Uganda (0.26 (SD 0.27)).

**Table 1:** Descriptive statistics Uganda and Honduras.

	Uganda N = 1392		Honduras N = 691
Age mean (SD)	37.05 (17.55)	Age mean (SD)	37.92 (15.23)
Education mean 1–9	3.22 (1.7)	Education mean 1–5	0.68 (0.72)
Gender (male)	47%	Gender (male)	47%
HH Assets quintile mean 1–5	3.21 (1.37)	HH income security mean 1–4	2.34 (0.79)
Marital status		Marital status	
Married	60%	Married	35%
Civil union	NA	Civil union	41%
Widowed	8%	Widowed	2%
Separated	4%	Separated	3%
Single	28%	Single	19%
Religion		Religion	
Protestant	71%	Protestant	NA
Catholic	24%	Catholic	75%
Evangelical	NA	Evangelical	17%
No religion	NA	No religion	8%
Other	5%	Other	NA
Ethnic group			
Banyankore	92%		NA
Bakiga	4%		NA
Baganda	3%		NA
Other	1%		NA
Household number	3.65 (1.87)	Household number	3.86 (1.95)
Respondent accepts IPV	29%	Respondent accepts IPV	24%
Household density	0.26 (0.27)	Household density	0.23 (0.19)
Mean proportion of ties same HH	0.21 (0.18)	Mean proportion of ties same HH	0.48 (0.44)
Mean proportion HH accept IPV	0.29 (0.38)	Mean proportion HH accept IPV	0.23 (0.34)
Mean proportion of HH married or civil union	0.56 (0.38)	Mean proportion of HH married or civil union	0.62 (0.32)
Mean proportion of HH male	0.51 (0.37)	Mean proportion of HH male	0.49 (0.32)

### Individual characteristics

Table 2 shows the association between demographic factors and IPV acceptance in the combined sample. Men were significantly less likely to accept IPV than women (Hindin, Kishor, & Ansara, 2008a). People of lower education and lower income were more likely to report IPV acceptance, while people who were older were less likely to report IPV acceptance. Although marital status, religion, ethnic group, and village were included in the model, they did not exhibit statistically significant associations with IPV acceptance at the p < 0.05 level. To test country-level differences, we removed ethnic group and village from the model (as both contained categories exclusive to one or more country). Consistent with our descriptive statistics, we found that people in Uganda were significantly more likely to accept IPV than people in Honduras.

**Table 2:** Demographic predictors of IPV acceptance in Uganda and Honduras, combined multivariate models.

	Combined Uganda and Honduras		
	OR	95% CI	P
Gender male	0.60	(0.48, 0.74)	0.00
Education	0.77	(0.68, 0.87)	0.00
Income	0.82	(0.74, 0.92)	0.00
Age	0.99	(0.98, 1.00)	0.00

Models also included marital status, religion, tribe, and village (not shown).

### Household level characteristics

For our main statistical analyses, we first tested the bivariate associations between our primary predictors and reported acceptability of IPV (Table 3). We found that household density, proportion of household that accepts IPV, mean household education, number of people in the household, and proportion of household that are male were all associated with IPV acceptance at the bivariate level. Factors with a p value of less than 0.10 in the bivariate models were incorporated into a set of multivariate models.

**Table 3:** Bivariate associations between individual and household predictors and individual IPV acceptance.

	Combined Uganda and Honduras		
	OR	95% CI	P
Proportion ties same HH	0.84	(0.76, 0.92)	0.00
Household density	0.88	(0.80, 0.98)	0.01
Number of HH members	0.95	(0.90, 1.00)	0.04
Proportion of HH that accepts IPV	1.49	(1.34, 1.64)	0.00
Proportion of HH that is male	1.18	(1.08, 1.30)	0.00
Proportion of HH that is married or in union	1.06	(0.96, 1.17)	0.27
Mean HH education	0.80	(0.72, 0.88)	0.00

**Fig. 1:**
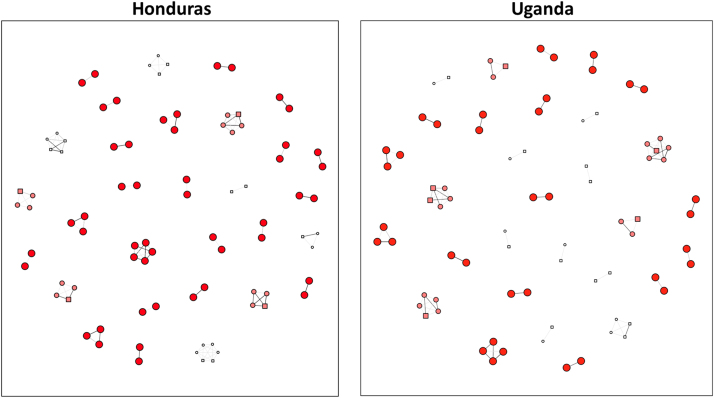
Depicts a random set of households in Honduras (left), and a random set of households in Uganda (right). Nodes (e.g. circles) represent individuals, lines represent relationships between the individuals within the same household, hence each cluster of nodes is a distinct household. Circular nodes do not accept IPV while square nodes do. Node color and node size are proportionate to household density: the large darkest red nodes are in high-density households while the small white nodes are in the low-density households. Note square nodes tend to be white, illustrating lower household cohesion for those individuals. Note also how the majority of those who accept IPV live in households in which at least one other household member also accepts IPV.

**Fig. 2:**
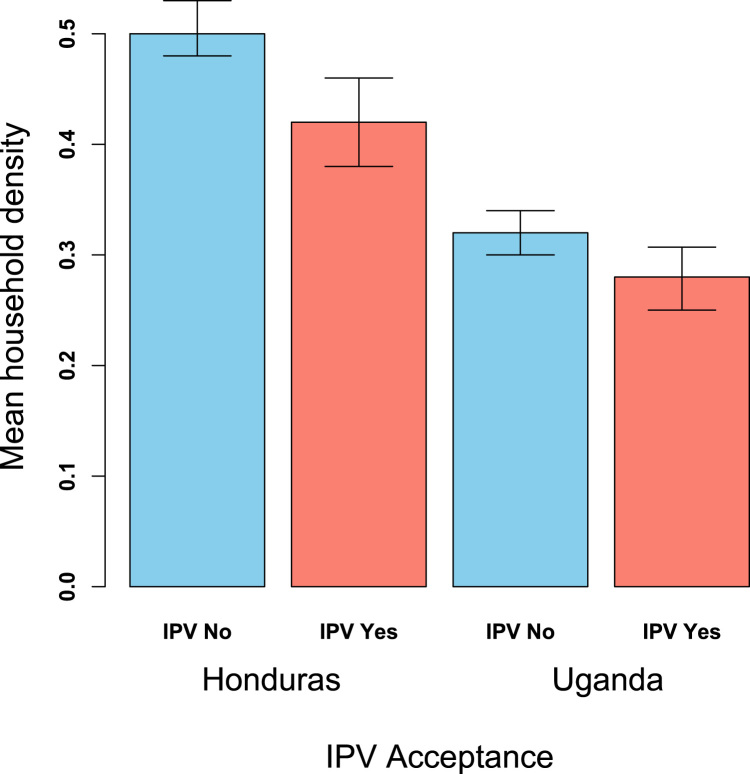
Illustrates the difference in mean household density for individuals who accept IPV versus those who do not. In both Honduras and Uganda, it is clear that household density is negatively associated with IPV acceptance.

Table 4 shows the results of a multivariate analysis of the association between our predictors of interest and individual IPV acceptance. This model excluded proportion of the household which accepts IPV, which had the strongest association with IPV acceptance in the bivariate models and which, as a proxy measure for household level norms, could affect the relationship between our cohesion variables and reported acceptance of IPV by individual subjects. We found that household density and mean education of the household retained significance in the multivariate models. The higher the density of the household, the less likely it was that an individual respondent would report acceptance of IPV: for each 1 standard deviation increase in household density the chance of any individual accepting IPV decreased by 15% (95% CI 5–24%). Fig. 1 depicts households in both Honduras and Uganda, with the larger darker colored red nodes in households with the highest density. Fig. 2 is a bar graph that depicts the difference in mean household density stratified by those who accept IPV and those who do not.,Similar to the density results, individuals who lived in households with higher levels of education were also less likely to accept IPV regardless of their own educational level. In fully-adjusted Model 2, we found that the proportion of the household that reported acceptance of IPV was associated with individual IPV acceptance. With each one standard deviation increase in proportion of household that accept IPV, the probability of any individual accepting IPV increases by 46% (95% CI 32%-51%). This model also showed that the associations between individual IPV acceptance and mean household education and household density, separately, were slightly attenuated after including proportion of household accepting IPV.

**Table 4:** Multivariate logistic regression showing the association between alter’s ipv acceptance and ego's ipv acceptance, combined Uganda and Honduras dataset.

	Model 1			Model 2		
	OR	95% CI	P	OR	95% CI	P
Proportion of HH that accepts IPV				1.46	(1.32, 1.61)	0.00
Proportion ties same HH						
Household density	0.85	(0.76, 0.95)	0.01	0.88	(0.79, .99)	0.03
Number of HH members	0.98	(0.92, 1.04)	0.55	0.98	(0.92, 1.05)	0.63
Proportion of HH that is male	1.04	(0.92, 1.18)	0.49	1.12	(0.99, 1.27)	0.08
Mean HH education	0.89	(0.79, 1.00)	0.00	0.92	(0.81, 1.04)	0.17
Sex	0.62	(0.47, 0.80)	0.00	0.62	(0.47, 081)	0.00
Education	0.80	(0.71, 0.91)	0.00	0.82	(0.72, 0.93)	0.00
Income	0.85	(0.76, 0.96)	0.01	0.86	(0.77, 0.97)	0.01
Age	0.99	(0.98, 1.00)	0.00	0.99	(0.98, 1.00)	0.00
AIC	2385			2331		

Models include marital status, religion, tribe and village (not shown).

As an exploratory analysis, for each individual, we also calculated the proportion of that individual’s nominated ties who lived in the same household, as an alternative measure to household density, which is a household-level measure. Table 5 shows the results of the same set of analyses but using the proportion of alters that are same household instead of household density in the models. The results are similar.

**Table 5:** Multivariate logistic regression showing the association between alter’s ipv acceptance and ego’s ipv acceptance, combined Uganda and Honduras dataset, testing proportion of household that accepts IPV.

	Model 1			Model 2		
	OR	95% CI	P	OR	95% CI	P
Proportion of HH that accepts IPV				1.47	1.33 1.62	0.00
Proportion ties same HH	0.85	(0.76, 0.95)	0.00	0.86	0.77 0.97	0.01
Household density						
Number of HH members	1.03	(0.97, 1.10)	0.30	1.03	0.97 1.09	0.39
Proportion of HH that is male	1.04	(0.92, 1.17)	0.53	1.12	0.99 1.27	0.09
Mean HH education	0.89	(0.79, 1.00)	0.05	0.92	0.82 1.04	0.19
Sex	0.60	(0.46, 0.78)	0.00	0.61	0.46 0.79	0.00
Education	0.80	(0.71, 0.91)	0.00	0.82	0.72 0.93	0.00
Income	0.85	(0.76, 0.96)	0.01	0.86	0.77 0.97	0.01
Age	0.99	(0.98, 1.00)	0.00	0.99	0.98 1.00	0.01
AIC	2385			2330		

**Fig. 3:**
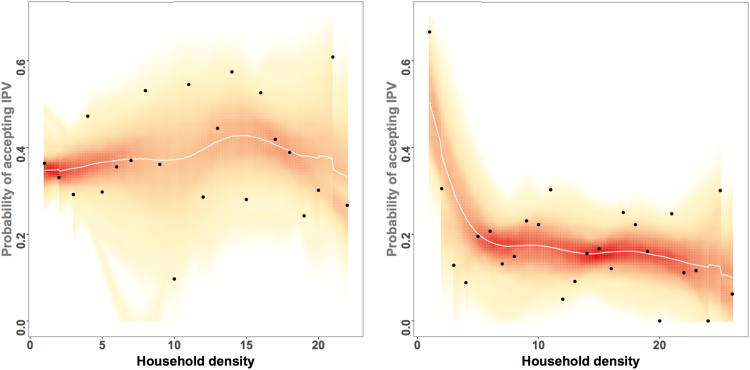
Shows the relationship between household density and IPV acceptance, stratified by those individuals in households with high acceptance of IPV compared to those in household with low acceptance of IPV.

Cohesive social networks can reinforce norms (whether protective or harmful), therefore it is likely that, while cohesion in general may decrease the likelihood that an individual accepts IPV, as we found in our results, this may change according to the overall level of IPV acceptance within the household. To understand this more deeply, we stratified our analysis to look at the association of household cohesion on IPV acceptance for those in households above or below the median proportion of household IPV acceptance within the sample. Table 6 shows the results of this analysis using household density as the primary predictor. In Model 1, for households that were below or equal to the median proportion that accept IPV, household density was significantly protective against individual IPV acceptance, as was living in a household with more members, and a household with a higher level of education. At higher levels of IPV acceptance (Model 2), however, we see that those associations are not observed. Statistical interaction tests (not shown) suggest that this difference is more pronounced in Honduras then in Uganda. Fig. 3 shows the relationship between household density and the probability that an individual accepts IPV for those in households above the median level of IPV acceptance, and for those below. There appears to be no relationship between household density and IPV acceptance for those in households with higher acceptance of IPV, while there is a clear relationship for those in households with lower acceptance. Further research is warranted to investigate this dynamic.

**Table 6:** Multivariate logistic regression showing the association between alter’s ipv acceptance and ego’s ipv acceptance, combined Uganda and Honduras dataset, stratified by household proportion of household that accepts IPV.

	Model 1 Lower proportion accepts IPV				Model 2 Higher proportion accepts IPV			
	OR	95% CI		P	OR	95% CI		P
Household density	0.83	0.72	0.96	0.01	0.97	0.80	1.17	0.76
Number of HH members	0.85	0.75	0.96	0.01	0.97	0.89	1.05	0.43
Proportion of HH that is male	1.16	0.97	1.38	0.11	1.04	0.85	1.26	0.72
Mean HH education	0.75	0.63	0.88	0.00	1.24	1.02	1.51	0.03
Sex	0.65	0.43	0.99	0.04	0.59	0.41	0.84	0.00
Education	0.77	0.63	0.93	0.01	0.88	0.73	1.06	0.18
Income	0.87	0.73	1.04	0.13	0.86	0.73	1.02	0.08
Age	0.99	0.98	1.00	0.04	0.99	0.98	1.00	0.03

To further understand these results, we investigated the household level factors associated with household density. We first calculated the mean household density for those households above the median proportion of household acceptance of IPV and for those below it. Households below the median proportion of household IPV acceptance had a mean-centered household density of 0.14 (95% CI 0.06–0.21) while households above had a mean-centered density of − 0.003 (95% CI − 0.06 to 0.05). Table 7 shows the results from a household-level linear regression estimating the relationship between household density and proportion of household that accepts IPV, proportion of household that is male, proportion of household that is married, and mean household education along with tribe and village. Here we see that household density is significantly and inversely related to the proportion of household that accepts IPV at the household level, inversely related to the proportion of household that is male, positively related to proportion of household that is married, and positively associated with mean household education level.

**Table 7:** Linear regressions analysis of household level predictors on household density.

	Combined Uganda and Honduras		
	Beta	Se	P
Proportion of HH that accepts IPV	− 0.08	0.02	0.00
Number in household	− 0.17	0.01	0.00
Proportion of HH that is male	− 0.04	0.02	0.04
Proportion of HH that is married	0.14	0.02	0.00
Mean HH education	0.09	0.02	0.00

We tested interactions by country (not shown) and found no significant differences between the association of household density and IPV acceptance by country but found a significant association with the proportion of household that accepts IPV and IPV acceptance. While proportion of household that accepts IPV was strongly associated with IPV acceptance in both countries, it was more strongly associated in Honduras compared to Uganda.

## Discussion

We describe the relationship between household cohesion, as measured through social ties within household members, and individual IPV acceptance in Honduras and Uganda. The more closely connected people are within a household, the less likely it is for an individual in that household to accept IPV, controlling for the overall acceptance of IPV within the household. Those in households more accepting of IPV were more likely to personally accept IPV. In stratified analyses, when household IPV acceptance was especially high, the benefit of household cohesion with respect to IPV was potentially attenuated, although further research is needed to investigate that preliminary evidence.

The household cohesion results provide possible evidence of the protective mechanism of strong family ties. More cohesive families might be more likely to enforce any existing familial norms against IPV in which case we would see a strong positive relationship between density and household acceptance of IPV in the households with higher overall levels of IPV acceptance. Alternately, families that are more cohesive may have better communication, more loving relationships, and an overall safer environment than less cohesive families (David, Olson, Douglas, & Sprenkle, 1983). Given previous work on the positive relationship between density and the maintenance of social norms (Shakya et al., 2014, Granovetter, 1983) it seems less likely that the relationship between cohesion and acceptance of IPV is simply one of social control, as the social control hypothesis would predict a positive association between density and IPV acceptance in household with high acceptance of IPV.

Densely connected networks of any sort are likely to reinforce positive or negative norms (Shakya et al., 2014, Granovetter, 1983). In this study, however, there was a lower likelihood that a person would accept IPV as the density of their household increased though this association potentially depended on the prevalence of IPV acceptance within the household. Notably, the protective association was possibly attenuated in households with high acceptance of IPV and the inclusion of household acceptance of IPV in the model diminished the significance of household density as a predictor. This dynamic is suggestive of counter-balancing mechanism by which the positive impact of family cohesion is in essence neutralized by the negative impact of high levels of acceptance of IPV. Also, our final analysis found that households with higher reported acceptance of IPV were associated with lower overall density. The fact that households with higher levels of IPV acceptance tend to also be those that are less densely connected suggests that density may reflect positive relationships within the family, and therein directly protect against approval of IPV through protective norms, rather than density simply reinforcing norms. Alternatively, the causal order could be reversed, in that higher prior levels of IPV among two members of a family may lead to lower density throughout the household. Finally, some other unmeasured factor in the household might have contributed to both IPV and decreased social ties.

Although household density was negatively associated with individual acceptance of IPV, the strongest predictor of individual acceptance was household acceptance of IPV. This finding is consistent with previous social network research in which the attitudes and behaviors of socially connected others are similar. In this case we are restricting those relationships to people who live within the same household. Our previous research in Honduras demonstrated that same household ties were the strongest predictor of shared attitudes towards IPV acceptance, suggesting that household members may be an important reference group for norms around IPV (Shakya et al., 2016). In this study, we find that household level factors are important in Uganda as well, although the magnitude of the effect was slightly smaller. This may be the because overall IPV acceptance is higher overall in Uganda than Honduras, making it slightly less sensitive to household-level factors.

It is important to note that, in this study, we did not have direct measures of either descriptive norms or injunctive norms, but in fact are inferring possible normative dynamics through measured attitudes. The fact that higher levels of IPV acceptance within a household is associated with individual IPV acceptance is a clue towards descriptive norms – the perception of something as prevalent and acceptable within a reference group increases the likelihood that any individual within that group will follow suit. That household density did not seem to reinforce higher levels of household IPV acceptance is a clue that acceptance of IPV is not necessarily normatively reinforced. However, the negative relationship between household density and individual IPV acceptance at lower levels of household IPV acceptance may suggest that when IPV is generally not acceptable, there is normative reinforcement to *not* accept IPV. Further research, with more robust measurement of social norms, is necessary to more clearly identify these dynamics.

Our study has limitations. First, the data are restricted to two villages in Honduras, and eight villages in Uganda. Thus, results might not be generalizable to other contexts. However, the consistency of our results across two international contexts suggests that interpretation of this information may be applicable outside of these specific settings. Second, analyses from both countries are cross-sectional. Therefore, we cannot infer causality, and the time-dependent process by which we could track changes in IPV attitudes with a change in household density is not possible. Finally, reports of IPV attitudes are self-report and can be subject to response bias, although we have used the standard DHS measures and so our results should be comparable to previous research conducted in this area. Moreover, attitudes are less prone to bias than behaviors. Finally, because of resource constraints, we were only able to survey 64% of the population in the Honduras sample.

## Conclusion

Here, we demonstrate the association between household characteristics and individual acceptance of IPV in 2 distinct geographic and cultural contexts. We find that not only does the proportion of an individual’s household that accepts IPV increase the likelihood that the individual accepts IPV, but individuals in more cohesive households are less likely to accept IPV. Future research on this topic should include measures of IPV perpetration, and longitudinal data so that changes in these dynamics can be tracked across time.

## Public health implications

Prevention of IPV is an important public health priority. While the best methods for IPV prevention are still being explored (Capaldi & Langhinrichsen-Rohling, 2012), some prior work has suggested that promotion of gender equality can reduce rates of IPV (McCloskey et al., 2005, Ackerson and S, 2008, Tsai, 2013). While this may be the case, it is important to take into consideration the target audience for these sorts of interventions. Our results provide evidence to suggest that gender equity and other preventive interventions may work best at the household level, where norms and attitudes on IPV are often enforced and reinforced. Furthermore, such intervention efforts may be most successful when they focus on fostering strong, healthy relationships within families, while promoting familial norms that serve to prevent violence against women in those families.

## Conflict of interests

Authors have no conflict of interests to disclose.

## References

[bib1] Ackerson L.K., Subramanian S.V. (2008). State gender inequality, socioeconomic status and intimate partner violence (IPV) in India: A multilevel analysis. Australian Journal of Social Issues.

[bib2] Ajzen I., Fishbein M. (1973). Attitudinal and normative variables as predictors of specific behavior. Journal of Personality and Social Psychology.

[bib3] Bell D.C., Cox M.L. (2015). Social norms: Do we love norms too much?. Journal of Family Theory Review.

[bib4] Borsari B., Carey K.B. (2003). Descriptive and injunctive norms in college drinking: A meta-analytic integration. Journal of Studies on Alcohol.

[bib5] Briceño-León R., Villaveces A., Concha-Eastman A. (2008). Understanding the uneven distribution of the incidence of homicide in Latin America. International Journal of Epidemiology.

[bib6] Capaldi D.M., Langhinrichsen-Rohling J. (2012). Informing intimate partner violence prevention efforts: Dyadic, developmental, and contextual considerations. Prevention Science.

[bib7] Centola D., Macy M. (2007). Complex contagions and the weakness of long ties. American Journal of Sociology.

[bib8] Cialdini R.B., Reno R.R., Kallgren C.A. (1990). A focus theory of normative conduct: Recycling the concept of norms to reduce littering in public places. Journal of Personality and Social Psychology.

[bib9] David H., Olson C.S.R., Douglas H., Sprenkle (1983). Circumplex model of marital and family systems: Vl. Theoretical update. Family Process.

[bib10] Garcia-Moreno C. (2005). WHO multi-country study on women’s health and domestic violence against women.

[bib11] Godfrey A.B. (2010). Household gender and resource relations: Women in the marketing arena of income generating crops in Uganda. Eastern Africa Social Science Research Review.

[bib12] Gorman-Smith D., Henry D.B., Tolan P.H. (2004). Exposure to community violence and violence perpetration: The protective effects of family functioning. Journal of Clinical Child and Adolescent Psychology.

[bib13] Granovetter M. (1983). The strength of weak ties: A network theory revisited. Sociological Theory.

[bib14] Hernandez P.M. (2002). Myth of Machismo: An everyday reality for Latin American women. The Thomas Law Review.

[bib15] Hindin M., Kishor S., Ansara D. (2008a). Intimate partner violence among couples in 10 DHS countries: Predictors and health outcomes. In Edited by International Calverton M. MD. 〈https://dhsprogram.com/pubs/pdf/AS18/AS18.pdf〉.

[bib16] Hindin M.J., Kishor S., Ansara D. (2008b). Intimate partner violence among couples in 10 DHS countries: Predictors and health outcomes. In *DHS analytical studies* . Edited by Inc., MI, *vol. no 18*. Calverton, MD.

[bib17] Kafumbe A.L. (2010). Women’s rights to property in marriage, divorce, and widowhood in Uganda: The problematic aspects. Human Rights Review.

[bib18] Kasozi A. (1994). Social origins of violence in Uganda, 1964–1985.

[bib19] Kennedy B.P., Kawachi I., Prothrow-Stith D., Lochner K., Gupta V. (1998). Social capital, income inequality, and firearm violent crime. Social Science Medicine.

[bib20] Kliewer W., Cunningham J.N., Diehl R., Parrish K.A., Walker J.M., Atiyeh C., Neace B., Duncan L., Taylor K., Mejia R. (2004). Violence exposure and adjustment in inner-city youth: Child and caregiver emotion regulation skill, caregiver–child relationship quality, and neighborhood cohesion as protective factor. Journal of Clinical Child and Adolescent Psychology.

[bib21] Kwagala B., Wandera S.O., Ndugga P., Kabagenyi (2013). Empowerment, partner’s behaviours and intimate partner physical violence among married women in Uganda. BMC Public Health.

[bib22] Latkin C.A., Forman V., Knowlton A., Sherman S. (2003). Norms, social networks, and HIV-related risk behaviors among urban disadvantaged drug users. Social Science & Medicine.

[bib23] Lomot R. (2013). Gender discrimination: A problem stunting Honduras' entire economy. Global Majority E-Journal.

[bib24] Marin A., Wellman B., Scott J., Carrington P. (2011). Social network analysis: An introduction. The SAGE handbook of social network analysis.

[bib25] McCloskey L.A., Williams C., Larsen U. (2005). Gender inequality and intimate partner violence among women in Moshi, Tanzania. International Family Planning Perspectives.

[bib26] Mirembe R., Davies L. (2001). Is schooling a risk? Gender, power relations, and school culture in Uganda. Gender and Education.

[bib27] Olsen A., Lovett R. (2016). Existing knowledge, practice, and responses to violence against women in Australian Indigenous communities: Key findings and future directions. Compass.

[bib28] Perkins J.M., Subramanian S., Christakis N.A. (2015). Social networks and health: A systematic review of sociocentric network studies in low-and middle-income countries. Social Science Medicine.

[bib29] Sambisa W., Angeles G., Lance P., Naved R., Thornton J. (2011). Prevalence and correlates of physical spousal violence against women in slum and nonslum areas of urban Bangladesh. Journal of Interpersonal Violence.

[bib30] Shakya H.B., Christakis N.A., Fowler J.H. (2014). Association between social network communities and health behavior: An observational sociocentric network study of latrine ownership in rural India. American Journal of Public Health.

[bib31] Shakya H.B., Hughes D.A., Stafford D., Christakis N.A., Fowler J.H., Silverman J. (2016). Intimate partner violence norms cluster within households: An observational social network study in rural Honduras. BMC Public Health.

[bib32] Shakya H.B., Christakis N.A., Fowler J.H. (2017). An exploratory comparison of name generator content: Data from rural India. Social Networks.

[bib33] Shakya H.B., Fleming P., Saggurti N., Donta B., Silverman J., Raj A. (2017). Longitudinal associations of intimate partner violence attitudes and perpetration: Dyadic couples data from a randomized controlled trial in rural India. Social Science & Medicine.

[bib34] Shakya H.B., Stafford D., Hughes D.A., Keegan T., Negron R., Broome J., McKnight M., Nicoll L., Nelson J., Iriarte E. (2017). Exploiting social influence to magnify population-level behaviour change in maternal and child health: Study protocol for a randomised controlled trial of network targeting algorithms in rural Honduras. BMJ Open.

[bib35] Statcompiler.

[bib36] Steinberg L., Lamborn S.D., Darling N., Mounts N.S., Dornbusch S.M. (1994). Over time changes in adjustment and competence among adolescents from authoritative, authoritarian, indulgent, and neglectful families. Child Development.

[bib37] Tsai A.C. (2013). Intimate partner violence and population mental health: Why poverty and gender inequities matter. PLoS Medicine.

[bib38] Vogt S., Mohmmed Zaid N.A., El Fadil Ahmed H., Fehr E., Efferson C. (2016). Changing cultural attitudes towards female genital cutting. Nature.

[bib39] WHO (2013). Global and regional estimates of violence against women: Prevalence and health effects of intimate partner violence and non-partner sexual violence. Geneva, Switzerland.

